# Identification of anti-tumoral feedback loop between VHLα and hnRNPA2B1 in renal cancer

**DOI:** 10.1038/s41419-020-02861-8

**Published:** 2020-08-11

**Authors:** Yanbin Liu, Hui Zhang, Xingzhi Li, Changming Zhang, Haide Huang

**Affiliations:** 1 Institute of Immunology and Molecular Medicine, Jining Medical University, Jining, China; 2 Department of Urological Surgery, Longgang District People’s Hospital of Shenzhen, Shenzhen, China; 3 Department of Biochemistry, Zhongshan School of Medicine, Sun Yat-sen University, Guangzhou, China

**Keywords:** Tumour-suppressor proteins, Urological cancer

## Abstract

Our previous study identified a novel VHLα isoform which negatively modulated hnRNPA2B1 expression and therefore influenced pyruvate kinase transcript splicing in renal cancer, while the regulation and initiation of alternative translation are largely unknown. Here we unraveled the CUG-mediated translation start of VHLα, which was subjected to the regulation by both eukaryotic initiator factor eIF2A and RNA helicase eIF4A. Unexpectedly, we found hnRNPA2B1 promoted VHLα alternative translation as well via direct interaction with its octadic pentamer region of VHL transcript. The N-terminal of VHLα was indispensable in mediating ubiquitination of hnRNPA2B1 at lysine residues 274 and 305. We further identified aberrant overexpression of c-myc as upstream oncogenic signaling to positively regulate hnRNPA2B1 transcription in renal cancer. Therefore, our data suggested an anti-tumoral feedback loop between VHLα and hnRNPA2B1.

## Introduction

VHL is well-recognized as essential tumor suppressor gene, and dysfunction of which intimately associates with malignancy-predisposing von Hipple-Lindau disease^1,2^. Assembling investigations uncover that loss-of-function of VHL via loss of alleles^3^, somatic mutation or epigenetic inhibition^4^ accounts for the majority of clear cell renal cell carcinomas (ccRCCs) incidence clinically^5^. Functionally, VHL constitutively associates with elongins B and C to form a relative stable ternary complex^6^, which further serves as adapter mediating the assembly of VCB-CUL2-RBX1^7,8^ with characteristic ubiquitin E3 ligase activity against α-subunit of hypoxia inducible factors (HIF1α and HIF2α)^9^. HIFα accumulation in the context of VHL-deficiency provokes array of oncogenic signaling, most of which link to acute or chronic hypoxic responses^10–12^. Therapeutics targeting VHL-HIF-VEGF pathway emerge as standard procedure in treatment of metastatic renal cell carcinoma^13^.

The coding region of VHL across three exons and encodes two proteins: VHL-24 and VHL-19. The latter initiates from an internal in-frame ATG codon and represents the predominant and conservative isoform^14–16^. Our pervious study uncovered the characteristic perinuclear distribution of VHL-19 complexing with the ribosomal large subunit 6 (RPL6), which may play critical roles in quality control of nascent peptides^17^. In addition, we identified a novel translation variant of VHL as well, termed VHLα, via alternative translation mechanism, which negatively regulates heterogenous nuclear ribonucleoprotein A2B1 (hnRNPA2B1) and therefore modulates pyruvate kinase transcription splicing^18^. However, the initiation site and regulation of VHLα alternative translation are still largely unknown. In view of well-recognized CUG-mediated alternative translation^19–21^, here we further pinpointed the initiation site of VHLα through serial synonymous mutations. The potential involvements of eukaryotic translation initiation factor 2A (eIF2A) which was previously indicated playing important roles in CUG-initiated translation^20^ are addressed in this study. We also attempt to systematic investigate the possible contributions of RNA helicases in VHLα translation with shRNA-mediated gene silencing.

Our previous study demonstrated that VHLα decreased hnRNPA2B1 at protein level which was efficiently blockaded by proteasome inhibitor, MG132, indicating the potential ubiquitination of hnRNPA2B1 by VHLα^18^. In this study, we set out to further identify the ubiquitination sites of hnRNPA2B1 and critical regions of VHLα responsible for protein interaction. Noting worthily, we also found that ectopic over-expression of hnRNPA2B1 stimulated short-term increase of VHLα translation (unpublished data), which suggested a potential post-transcriptional regulation of VHLα by hnRNPA2B1 in turn. As a ubiquitous RNA binding protein^22^, the essential biological roles of hnRNPA2B1 are increasingly unraveled. Here we unraveled the mutual regulation between VHLα and hnRNPA2B1, which constitutes feedback loop in malignancy of ccRCC.

## Materials and methods

### Cell culture and transfection

Human kidney cell HEK293T and ccRCC cell lines 786–0, 769-p, ACHN, A498, A704, Caki-1, Caki-2 and RCC4 were obtained from the American Typical Culture Collection (ATCC, NY, USA). Cell identities were authenticated by short tandem repeat (STR) profiling and regularly examined mycoplasma contamination by PCR method. HEK293T cells were cultured in DMEM high glucose and other cells in RPMI modified medium, all mediums were supplemented with 10% fetal bovine serum (FBS) and 1% penicillin/streptomycin. Cell culture was maintained in humidified CO_2_ incubator (5%). Transfection was performed with Lipofectamine 2000 (Invitrogen, MO, USA) in accordance with manufacturer. All shRNAs were purchased from Origene (Beijing, China) and overexpressing plasmids for VHL, hnRNPA2B1, c-myc, HA-eIF2A, HA-eIF4A were ordered from Vigenebio (Jinan, China).

sheIF2A-1: 5′-CAGCCTTACACTACTTCTAAAGATGGCAC-3′;

sheIF2A-2: 5′-TACAAGGTGGCTGTCTATGTTCCAGGAAG-3′;

shNT: 5′-GCACTACCAGAGCTAACTCAGATAGTACT-3′;

shDed1: 5′-ACTCATCCGAAATGGACGTGACTTCTTAT-3′;

shDHX29: 5′-ATTGAAGATGCCATGACCAATACACTCTT-3′;

shRHA: 5′-ACCGAGGAGCCAACTTGAAGGATTACTAC-3′;

shVASA: 5′-GAGGACTCCATCTTTGCACATTATCAGAC-3′;

sheIF4A-1: 5′-AGCAGCGAGCCATTCTACCTTGTATCAAG-3′;

sheIF4A-2: 5′-ATTGGCTCAGCAGATACAGAAGGTGGTCA-3′;

shc-myc: 5′-GAGGATATCTGGAAGAAATTCGAGCTGCT-3′;

shhnRNPA2B1: 5′-GCTACGGAGGTGGTTATGACAACTATGGA-3′.

### Clinical samples

Totally, 30 ccRCC tumor samples were collected from Longgang District People’s Hospital from 20015 to 2019 during radical surgery, and pathologically examined by three independent pathologists. The fresh tissues were flash frozen in liquid N2 until use. The written informed consents were acquired from all enrolled subjects and this study was approved by Institutional Ethics Committees of Longgang District Hospital.

### Western blots

Cell lysates were prepared in RIPA lysis buffer on ice and debris were completely removed by refrigerated centrifugation. One microgram of plasmids were used for transfection and protein and was harvested 24 h later unless specified. For drug treatments in 786–0 cells, 100 μM Aurin tricarboxylic acid was applied for 0, 6, 12, 24 h, different dosage of Acriflavine (0, 10, 20, 30 μM) was applied for 4 h, Pateamine A (15 μM), Hippuristanol (5 μM) and Silvestrol (20 nM) were applied individually for 0, 12, and 24 h, respectively. In induced expression of hnRNPA2B1, Doxycycline (0, 0.1, 0.2, 1, 2 μM) was applied for 4 h or 0.5 μM of Doxycycline was applied for 0, 6, 12, 18, and 24 h, respectively. All proteins were resolved with 12% SDS-PAGE and transferred onto PVDF membrane (Millipore, MO, USA) on ice. 5% skim milk in TBST buffer was used for brief blocking and followed by incubation with primary antibody (VHLα antisera were prepared in mice as previously described^18^; rabbit anti-β-actin, 1:2000, #4967; rabbit anti-VHL, 1:1000, # 68547; rabbit anti-eIF2A, 1:1000, #5324; rabbit anti-eIF4A, 1:1000, #2425 from Cell Signaling Technology, MA, USA; mouse anti-FLAG, 1:2500, F3165; mouse anti-HA, 1: 5000, H9658; rabbit anti-hnRNPA2B1, 1: 2500, HPA001666; rabbit anti-Myc tag, 1: 500, 06–549; rabbit anti-c-myc, 1: 1000, C3956 from Sigma, MO, USA) overnight at 4 °C. After wash for 30 min, the membrane was hybridized with HRP-conjugated secondary antibodies (goat anti-rabbit, 1: 5000, #7074; horse anti-mouse, 1: 5000, #7076; Cell Signaling Technology, MA, USA) for another hour. The blots were visualized using the enhanced chemiluminescence kit (ECL, Millipore, MO, USA).

### Real-time PCR

Total RNA extraction from either indicated tissues samples or cells was performed with TRIzol method (Invitrogen, MA, USA) following the provider’s protocol. RNA quality was examined with Bioanalyzer 2100 (Agilent, CA, USA) prior to use. Reverse transcription with 1 μg RNA was performed with High Capacity cDNA Reverse Transcription Kit (Applied Biosystems, CA, USA). The SYBR Green Real-Time Master Mixes (ThermoFisher, MA, USA) was used for quantitative PCR on CFX96 Touch Real-Time PCR Detection System (Bio-Rad, CA, USA). The primer sequences used were listed as follows:

eIF2A Forward primer: 5′-CCGCTCTTGACAGTCCGAG-3′;

eIF2A Reverse primer: 5′-GCAGTAGTCCCTTGTTAGTGACA-3′;

eIF4A Forward primer: 5′-AAGGCGTCATCGAGAGTAACT-3′;

eIF4A Reverse primer: 5′-ATGTGGCCGTTTTCCCAGTC-3′;

VHL-24 Forward primer: 5′-GCAGGCGTCGAAGAGTACG-3′;

VHL-24 Reverse primer: 5′-CGGACTGCGATTGCAGAAGA-3′;

VHL-19 Forward primer: 5′-GGAGCCTAGTCAAGCCTGAGA-3′;

VHL-19 Reverse primer: 5′-CATCCGTTGATGTGCAATGCG-3′;

VHLα Forward primer: 5′-CGCGTTCCATCCTCTACCGA-3′;

VHLα Reverse primer: 5′-TCTTCGACGCCTGCCTCCT-3′;

c-myc Forward primer: 5′-GGCTCCTGGCAAAAGGTCA-3′;

c-myc Reverse primer: 5′-CTGCGTAGTTGTGCTGATGT-3′;

hnRNPA2B1 Forward primer: 5′-TGGAGGTAGCCCCGGTTATG-3′;

hnRNPA2B1 Reverse primer: 5′-GGACCGTAGTTAGAAGGTTGCT-3′;

β-actin Forward primer: 5′-CATGTACGTTGCTATCCAGGC-3′;

β-actin Reverse primer: 5′-CTCCTTAATGTCACGCACGAT-3′.

### Immunohistochemistry (IHC)

The ccRCC tissues were embedded in paraffin blocks and sectioned into 5 μm slices following the standard protocol. The slides were then deparaffinized in xylene for 5 min by two times and rehydrated in absolute alcohol for 3 min by two times, 95%, 70%, 50% alcohol for 3 min each. The IHC staining procedure was performed with Biotin-Streptavidin HRP Detection Systems (ZSGB-BIO, Beijing, China) following the manufacturer’s protocol. Endogenous peroxidase activity was blockaded with 3% H_2_O_2_ solution in methanol at room temperature for 10 min. Antigen retrieval was achieved by heating in citrate buffer (10 mM, pH 6.0) using microwave. After blocking with 10% FBS, tissue slides were applied with indicated primary antibody (VHLα antisera, 1:20. Rabbit anti-myc, 1:25, HPA055893; rabbit anti-hnRNPA2B1, 1: 50, HPA001666; rabbit anti-eIF2A, 1:200, HPA036256 from Sigma, MO, USA. Rabbit Anti-eIF4A, 1:100, ab31217 from Abcam, Cambridge, UK) in humidified chamber at room temperature for 1 h. The biotin-labeled secondary antibody was then incubated for 30 min and HRP-streptavidin conjugates were applied for another 15 min at room temperature. Chromogenic reaction was performed with diaminobenzidine (DAB) reagent. Representative images were acquired under DMi8 Inverted Microscope (Leica, Wetzlar, Germany).

### RNA pulldown

RNA pulldown assay was performed with Pierce Magnetic RNA-Protein Pull-Down Kit (ThermoFisher, MA, USA) in accordance with manufacturer’s instruction. All biotin-labeled RNA fragments were synthesized by Synobio-Tech (Suzhou, China). VHL-19, VHL-24, and VHLα transcripts were prepared with MEGAscript T7 Transcription Kit (ThermoFisher, MA, USA) following the manufacturer’s manual and biotin-labeled with Pierce RNA 3′ End Biotinylation Kit (ThermoFisher, MA, USA). Briefly, biotin-labeled RNA (100 pmol) was captured by 50 μL of Streptavidin Magnetic Beads for 30 min at room temperature, and incubated with 100 μg of cell lysate in RIPA lysis buffer for 60 min at 4 °C with agitation. The pull-downed protein species were eluted with Elution buffer for 0 min at 37 °C with agitation, and subjected to western blots analysis.

### RNA immunoprecipitation (RIP)

RNA-IP assay was performed with Imprint RNA Immunoprecipitation Kit (Sigma, MO, USA) according to the manufacturer’s manual. Briefly, 5 μg of hnRNPA2B1 antibody was immobilized on 20 μL of Protein A Magnetic Beads for 30 min at room temperature, then incubated with cell lysates (10^6^ cells/20 μL lysis buffer) overnight at 4 °C with rotation. RNA was recovered with TRIzol (500 μL per RIP reaction) methods and resuspended in 20 μL of RNase-free water. Enrichments of target transcripts were analyzed by real-time PCR as previously described.

### Co-immunoprecipitation (Co-IP)

Indicated cells were collected in ice-cold PBS with scraper and lysed in 500 μL of IP lysis buffer (50 mM HEPES, pH 7.5, 150 mM NaCl, 1 mM EDTA, 2.5 mM EGTA, 0.1% (w/v) Tween20, 1 mM dithiothreitol, 1 mM NaF and 100 µM PMSF) on ice for 30 min. Protein concentration was determined with BCA Protein Assay Kit (ThermoFisher, MA, USA), and 2 mg of protein (in 500 μL of lysis buffer) was incubated with 2 μg of rabbit IgG for 1 h at 4 °C for clearance purpose, followed by incubation with anti-FLAG M2 Magnetic Beads overnight at 4 °C with agitation. Immunoprecipitated complex was recovered with SDS-PAGE sample buffer and analyzed by western blots.

### Immunofluorescence

786–0 cells were plated on cover slips and cultured overnight for attachment. Cells were fixed with 4% PFA for 15 mins and permeabilized with 0.2% Triton X-100 for 10 min. Anti-hnRNPA2B1 (HPA065537, 1:400, Sigma, MO, USA) and VHLα antisera (1:20) were then incubated at 4 °C overnight after brief blocking with 5% BSA, and followed by hybridization with fluorescent secondary antibody (anti-rabbit 488, 1:200; anti-mouse 594, 1:200; ThermoFisher, MA, USA). The coverslips were mounted with ProLong Gold Antifade Mountant with DAPI (ThermoFisher, MA, USA) on glass slides. The images were acquired with confocal microscope (Leica, Wetzlar, Germany).

### Chromatin immunoprecipitation (ChIP)

The indicated cells (10^6^) were first fixed with formaldehyde (1%) at room temperature for 10 min and lysed in 0.5 mL of ice-cold lysis buffer supplemented with Protease Inhibitor Cocktail (Roche, Basel, Switzerland) for 15 min. Cell nuclear were collected in 0.5 mL of nuclear lysis buffer and subjected to sonication to shear crosslinked DNA to 200–1000 bp fragments. After centrifugation, the supernatant was incubated with 2 μg of c-myc antibody (AHO0062, ThermoFisher, MA, USA) and 20 μL of well-dispersed protein G magnetic beads overnight at 4 °C with rotation. Immunoprecipitated complex was collected, and enriched DNA fragments were recovered by protease K digestion at 62 °C for 2 h with shaking and purified using spin column. The presence of hnRNPA2B1 promoter fragments were analyzed by real-time PCR. The primer sequences used for ChIP analysis were indicated in figure legend.

### Luciferase reporter assay

The exponential HEK293T cells were seeded into 6-well plate (5 × 10^5^/well). The hnRNPA2B1 promoter (~1 kb) luciferase reporter plasmid was ordered from GeneCopoeia (Guangzhou, China). Point-directed mutagenesis was employed to generate putative c-myc-recognizing site mutants. The luciferase plasmids (1 μg) were co-transfected with either empty control or c-myc-expressing plasmids (1 μg) into HEK293T cell (6-well plate) for 24 h, and relative luciferase activities were measured with Bright-Glo Luciferase Assay System (Promega, WI, USA) on BioTek Synergy 2 Multi-Mode Microplate Reader (BioTek, VT, USA). The mutagenesis PCR primer sequences were listed as below:

Forward: 5′-TGCGCGACCGTTTCTTCGCACCCTTGCGCCAAGGCAG-3′;

Reverse: 5′-CTGCCTTGGCGCAAGGGTGCGAAGAAACGGTCGCGCA-3′.

### Cell counting kit-8 (CCK-8) assay

The indicated cells at log phase were inoculated in 96-well plate (1000 cells/well) and cultured up to 4 days. Cell viability was determined with CCK-8 kit (Dojindo, Dalian, China) following the manufacturer’s instruction at day 0, 1, 2, 3, and 4, respectively. Ten microliter of CCK-8 solution was added into each well and incubated for 2 h in CO2 incubator. Absorption was measured with microplate reader (BioTek, VT, USA).

### Colony formation

The indicated cells (10^4^/well) were plated in 6-well plate and subjected to continuous culture in regular medium for up to 10 days. Fresh culture medium was replaced every 3 days. After fixation with 4% PFA (15 min at RT) and staining with crystal violet (10 min at RT), the resultant colonies were counted under microscopic and images were captured with DMi8 Inverted Microscope (Leica, Wetzlar, Germany).

### Transwell assay

The polycarbonate membrane cell culture inserts (Coring, NY, USA) were employed to measure cell invasive capacity. The single-cell suspension prepared from indicated cells via trypsin digestion (cells/μL) and 100 μL of cells (in serum-free medium) were seeded into the upper inserts with 750 μL of complete medium supplied in lower compartments. After cultured for 12 h, the invaded cells were fixed with 4% PFA for 15 min and stained with crystal violet (0.25%) for 30 min. The images were captured, and cells were counted under microscope.

### Wound healing assay

Wound healing assay was employed to evaluate cell migrative capacity. The indicated cells (2 × 10^5^) were inoculated in 6-well plate and allowed for attachment overnight. The scratch was created with sterile tips and detached cells were aspirated with PBS wash. Cells were cultured up to 48 h and gap closure was continuously monitored under light microscope.

### Xenograft tumor

The indicated cells (10^6^ cells) were prepared as single-cell suspension into 100 μL of PBS: Matrigel Basement Membrane Matrix (1: 1, Corning, NY, USA), and inoculated into the lower flanks of BALB/c Nude (4-weeks-old, Vitalriver, Beijing, China). The xenograft tumor growth was continuously monitored up to 18 days post-inoculation and mice were sacrificed by cervical dislocation. Tumor volume was estimated as (Length × Width^2^)/2. Mice were equally divided into six groups: empty vector, c-myc-overexpressing, c-myc-overexpressing plus hnRNPA2B1-silencing, c-myc-overexpressing plus VHLα-overexpressing, hnRNPA2B1-overexpressing, hnRNPA2B1-overexpressing plus VHLα-overexpressing, 5 mice for each group. The animal study was approved by the Institutional Animal Care and Use Committee of Jining Medical University. Experimental mice were housed in specific pathogen-free (SPF) environment and all procedures were in strict compliance with NIH guideline.

### Statistical analysis

Data presented in this study were analyzed and processed using Prism 8 (GraphPad, CA, USA). Unpaired student *t*-test was employed for statistical comparison between groups. The *p* value was calculated and *p* < 0.05 was considered as statistically significant.

## Results

### eIF2A and eIF4A involve in VHLα alternative translation

**Fig. 1:**
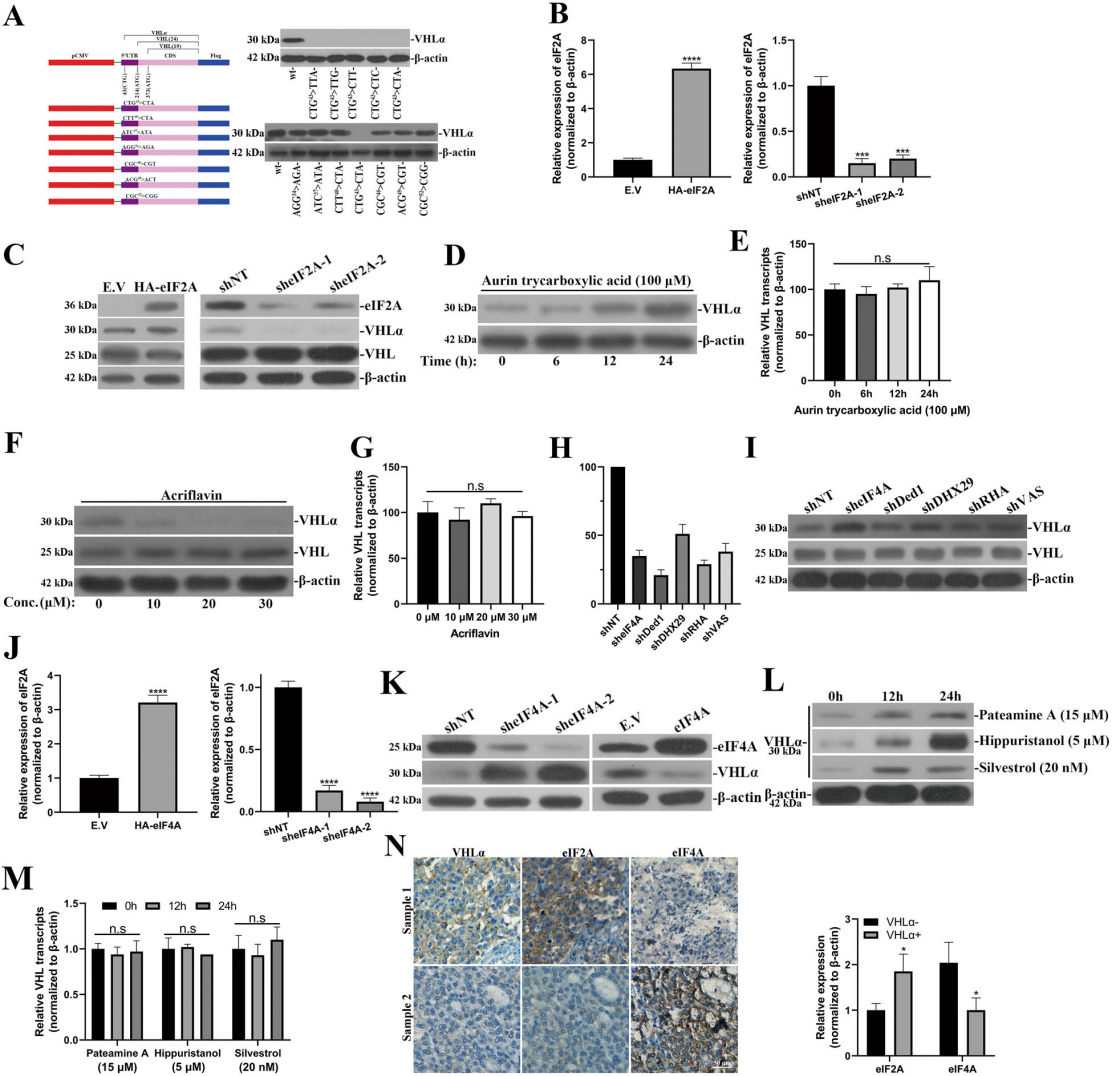
eIF2A and eIF4A involve in VHLα alternative translation. **a** Immunoblots analysis of ectopic VHLα in 293T cells in response to synonymous mutations introduced at CTG^43^ (left) and neighboring codons (AGG^34^ > AGA, ATC^37^ > ATA, CTT^40^ > CTA, CGC^46^ > CGT, ACG^49^ > ACT and CGC^52^ > CGG, right). **b** Quantitative PCR measurements of relative expression of eIF2A in established stable cells (786–0 with empty vector (E.V) or HA-eIF2A, 769-p with shNT, sheIF2A-1 and sheIF2A-2). **c** Immunoblots analysis of VHLα in response to eIF2A overexpression (left) or knockdowns (right) in 293T. **d** Time course of VHLα induction in 293T cells by aurin tricarboxylic acid (ATA, 100 αM) treatments for 0, 6, 12, 24 h. **e** VHL mRNA transcript levels were determined by real-time PCR in ATA-treated 293T cells at 0, 6, 12, and 24 h. **f** Immunoblots analysis of VHLα and VHL in response to different concentrations of scriflavin treatments (0, 10, 20, 30 M) for 4 h in 293T cells. **g** VHL mRNA transcript levels were determined by real-time PCR in acriflavin-treated 293T cells as indicated in **f**. **h** Knockdown efficiencies of DNA helicases including eIF4A, Ded1, DHX29, RHA, and VASA were evaluated by real-time PCR. **i**. VHLα protein was examined in DNA helicase-deficient 293T cell by western blots. **j** Establishment of eIF4A-overexpressing (HA-eIF4A) and eIF4A-silencing (sheIF4A-1 and sheIF4A-2) cell lines was validated by real-time PCR. **k** VHLα protein alterations upon eIF4A overexpression and knockdown were evaluated by immunoblotting in 293T cells. **l** Relative VHLα proteins were examined in 293T in response to eIF4A-specific inhibitors for 0, 12, and 24 h (pateamine A, 15 μM; hippuristanol 5 μM; silvestrol 20 nM). **m** Consistent VHL transcript abundance in response to eIF4A inhibitors treatments as described in **l**. was measured by real-time PCR. **n** Representative IHC images of VHLα− and VHLα+ renal tumors (left) and analysis of relative expression of eIF2A and eIF4A regarding VHLα status by real-time PCR. Data are presented as mean ± S.D for three independent experiments. n.s., no significance; *P < 0.05; **P < 0.01; ***P < 0.001; ****P < 0.0001.

Our previous study first uncovered the alternative translation of VHL transcript and identified a novel protein isoform named VHLα^18^. However, the alternative translation initiation codon was still to be defined and the molecular mechanism underlying regulation of alternative translation of VHL transcript was largely elusive. In view of the widely recognized leucine-initiator tRNA-mediated translation start at CUG codon, we hypothesized that similar mechanism might operate in control of VHLα translation after close inspection of the 5′ region of VHL transcript. All 5 synonymous mutations introduced to CTG^43^ codon completely abolished VHLα expression (Fig. 1a). On the contrary, synonymous mutations affecting the neighboring codons including AGG^43^, ATC^37^, CTT^40^, CGC^46^, ACG^49^, and CGC^52^ manifested none of noticeable impacts on VHLα translation (Fig. 1a, lower). Via point-directed mutations, we provided evidence the alternative translation of VHLα initiated from CTG^43^ codon. We then further clarified the potential involvement of eukaryotic translation initiation factor 2 A, eIF2A, in this scenario, which was previously suggested playing crucial roles in mediating CUG-initiated translation. To this end, we established ectopic eIF2A-overexpressing (Fig. 1b, left) and eIF2A-silencing (Fig. 1b, right) cell lines and assessed the influences on VHLα translation. As shown in Fig. 1c, endogenous VHLα was increased in eIF2A-proficient cells in comparison with empty vector control, while significantly inhibited by shRNA-mediated knockdown of eIF2A. This result suggested the indispensable role of eIF2A contributing to VHL alternative translation. Furthermore, we employed aurin tricarboxylic acid (ATA) to efficiently suppress translation start at the AUG codon and enhance CUG-mediated initiation. ATA treatment significantly promoted VHLα expression in a time-dependent manner (Fig. 1d), while showed no evident effects on VHL transcript levels (Fig. 1e). On the other hand, acriflavine greatly compromised VHLα expression in a dose-dependent manner (Fig. 1f), which was consistent with previous report about inhibitory action of acriflavine on CUG initiation^20^. Similarly, the transcript abundance of VHL was not altered by acriflavine treatment (Fig. 1g), which implicated a regulation at post-transcriptional level.

As we described previously, the octadic pentamers of VHL transcript highly likely led to formation of complex secondary structure, which hindered translation scanning and necessitated RNA helicase activities for efficient protein synthesis. To clarify the potential involvements of RNA helicases in regulation of VHL translation, we effectively silenced eIF4A, Ded1, DHX29, RHA, and VASA individually (Fig. 1h). VHLα was slightly upregulated only in the context of eIF4A knockdown (Fig. 1i). To further solidify our observation, we generated eIF4A-overexpressing cells and specifically silenced eIF4A with two independent shRNAs (Fig. 1j). VHLα protein was significantly induced by eIF4A-depletion and reduced by ectopic eIF4A (Fig. 1k). We further employed eIF4A-specific inhibitors including pateamine A^23^, hippuristanol^24^ and silvestrol^25^, all of which showed stimulatory effects on VHLα translation in a time-dependent manner (Fig. 1l). To exclude possible influences of eIF4A inhibitors on VHL transcription, we also analyzed mRNA and confirmed the consistent level of VHL transcripts (Fig. 1m). Therefore, our data suggested that eIF4A played important roles in promoting VHL translation and therefore suppressed VHLα expression, which critically depended on its RNA helicase activities. In addition, we analyzed relative expression of eIF2A and eIF4A regarding VHLα status in clinical tumors by both IHC analysis and real-time PCR, and noticed significant upregulated eIF2A and suppressed expression of eIF4A in VHLα-positive samples (Fig. 1n). Taken together, our results identified the CUG-initiated VHLα translation, which was subjected to modulation by both eIF2A and eIF4A.

### Direct association between hnRNPA2B1 and octadic pentamer region of VHLα transcript

**Fig. 2:**
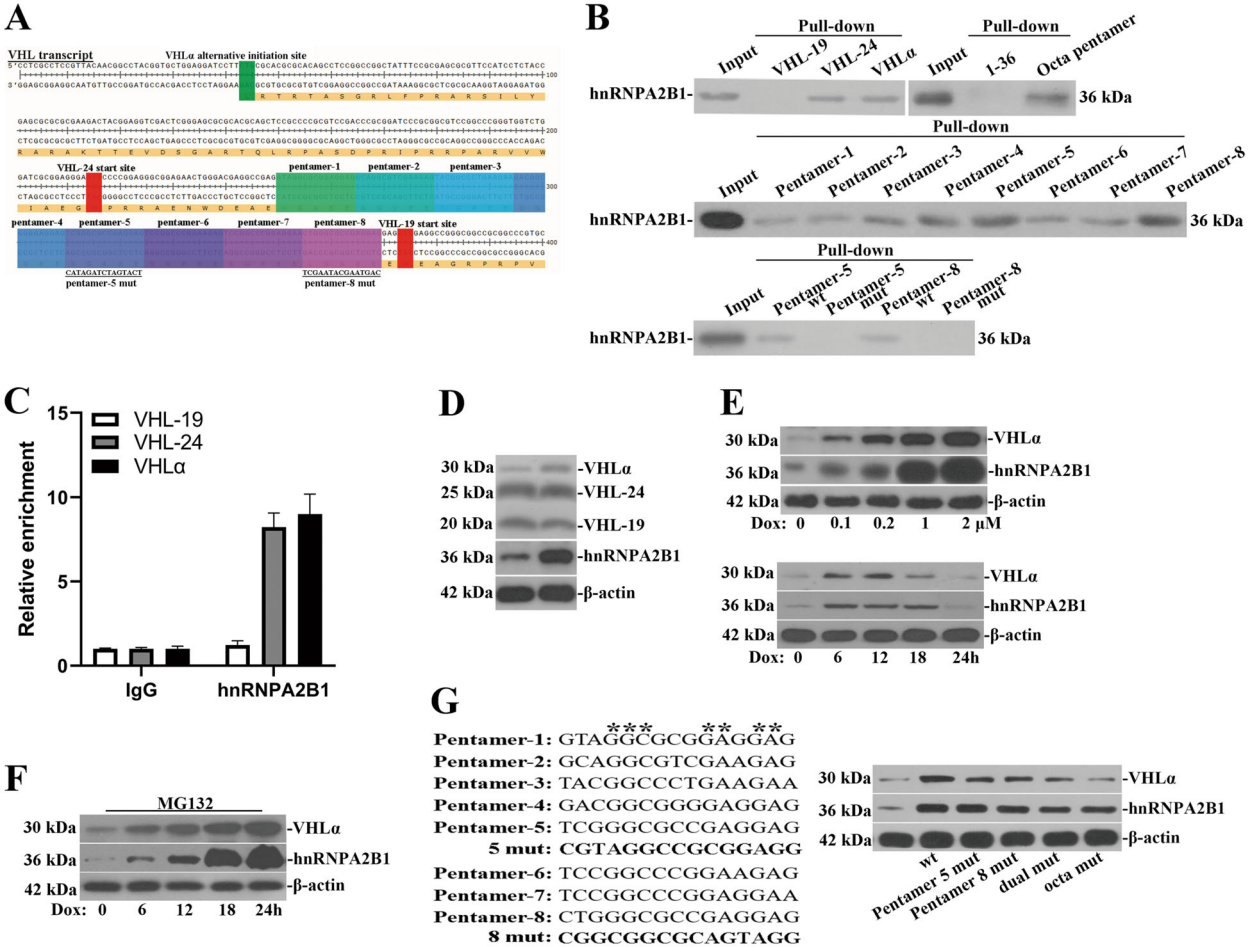
hnRNPA2B1 directly associates with and modulated VHLα mRNA translation. **a** Diagrammatic illustration of 5’ region of VHL transcript with initiation codons and octadic pentamers highlighted in colors. **b** Western blots detection of hnRNPA2B1 in pull-down complex with biotin-labeled RNA species as indicated. The mutant sequences of pentamer-5 and pentamer-8 were shown in **a**. **c** RNA-IP performed with either IgG control or anti-hnRNPA2B1 antibody and relative enrichments of VHL-19, 24, and VHLα were analyzed by real-time PCR. **d** Western blots analysis of VHLα along with VHL-19 and 24 proteins in both empty vector and hnRNPA2B1-overexpressing plasmids-transfected 293T cells. **e** Concentration-(0, 0.1, 0.2, 1, and 2 μM for 4 h) and time-dependent (0.2 μM for 0, 6, 12, 18 and 24 h) changes of VHLα protein in doxycycline-inducible hnRNPA2B1-overexpressing 293T cells were investigated by western blots. **f** Western blots analysis of VHLα in doxycycline-induced hnRNPA2B1-overexpressing 293T cells in the presence of proteasome inhibitor, MG132 (5 μM). **g** Alignment of eight pentamer sequences and scramble mutations, and the conservative bases were highlighted with stars. The potential impacts of mutation introduced into pentamer-5 and pentamer-8 on VHLα protein in the context of hnRNPA2B1 overexpression was evaluated by western blots in 293T cells. n.s., no significance; *P < 0.05; **P < 0.01; ***P < 0.001; ****P < 0.0001.

Our previous study demonstrated that hnRNPA2B1 was significantly decreased by VHLα other than other isoforms, which was blockaded by proteasome inhibitor, MG132, and suggested the involvement of ubiquitination/proteasome system-mediated protein degradation^18^. We also noticed that MG132 treatment caused increase of VHLα in addition to accumulation of hnRNPA2B1, which implicated the possible feedback regulation on VHLα. In view of the ubiquitous affinity of hnRNPA2B1 to RNA species, here we set out to clarify the physical association between hnRNPA2B1 protein and VHL transcript. The characteristic features of 5′ VHL transcript were illustrated in Fig. 2a, with start sites of VHL-19, VHL-24, VHLα and octadic pentamers were highlighted in colors, and pentamer-5 and pentamer-8 mutant sequences were provided as well. The biotin-labeled pulldown assay demonstrated that hnRNPA2B1 complexed with both VHL-24 and VHLα transcript except for VHL-19, suggesting the recognizing site might locate between VHL-19 and VHL-24 start sites (Fig. 2b, upper left). The fragmentation RNA pull-down assay showed that octadic pentamer was responsible for interacting with hnRNPA2B1 (Fig. 2b, upper right). Therefore, we examined the eight pentamer sequences individually and found all of them showed affinity to hnRNPA2B1, while among of which pentamer-5 and pentamer-8 were the most significant ones (Fig. 2b, middle). Scramble mutations completely abolished the presence of hnRNPA2B1 in pentamer-5 and pentamer-8 pull-downed protein species (Fig. 2b, lower). We also performed RNA immunoprecipitation with hnRNPA2B1 antibody and observed significant enrichments of VHL transcripts which were detected by VHL-24 and VHLα-specific primers (Fig. 2c). These data provided solid evidences supporting the direct interaction between hnRNPA2B1 and VHL transcripts.

To further address the possible regulation of VHLα translation by hnRNPA2B1 in return, we ectopically over-expressed hnRNPA2B1 for a short-term and noticed increase of VHLα protein (Fig. 2d). In doxycycline-induced overexpressing system, VHLα increased with Dox in a dose-dependent manner (Fig. 2e). However, long-term exposure to Dox led to decreases of both VHLα and hnRNPA2B1 despite of increases at early stage (Fig. 2e, lower), which hinted that accumulated VHLα promoted hnRNPA2B1 ubiquitination and degradation, and which eventually compromised the stimulatory effects on VHLα translation. Addition of MG132, which stabilized hnRNPA2B1 and therefore continuously stimulated VHLα translation up to 24 h in our system (Fig. 2f). This stimulation was greatly dependent on recognition of pentamer sequences especially pentamer-5 and pentamer-8 by hnRNPA2B1, which was consolidated by the observation that scramble mutations disrupting either pentamer-5 or pentamer-8 compromised VHLα translation. Dual mutations of both pentamer-5 and pentamer-8 greatly suppressed VHLα expression, while disruption of all eight pentamer sequences with scramble mutation almost completely abrogated the regulation of VHLα by hnRNPA2B1 (Fig. 2g). Taken together, our data suggested that hnRNPA2B1 positively regulated VHLα translation via direct association with its octadic pentamer region.

### VHLα associates with and mediates ubiquitination and degradation of hnRNPA2B1

**Fig. 3:**
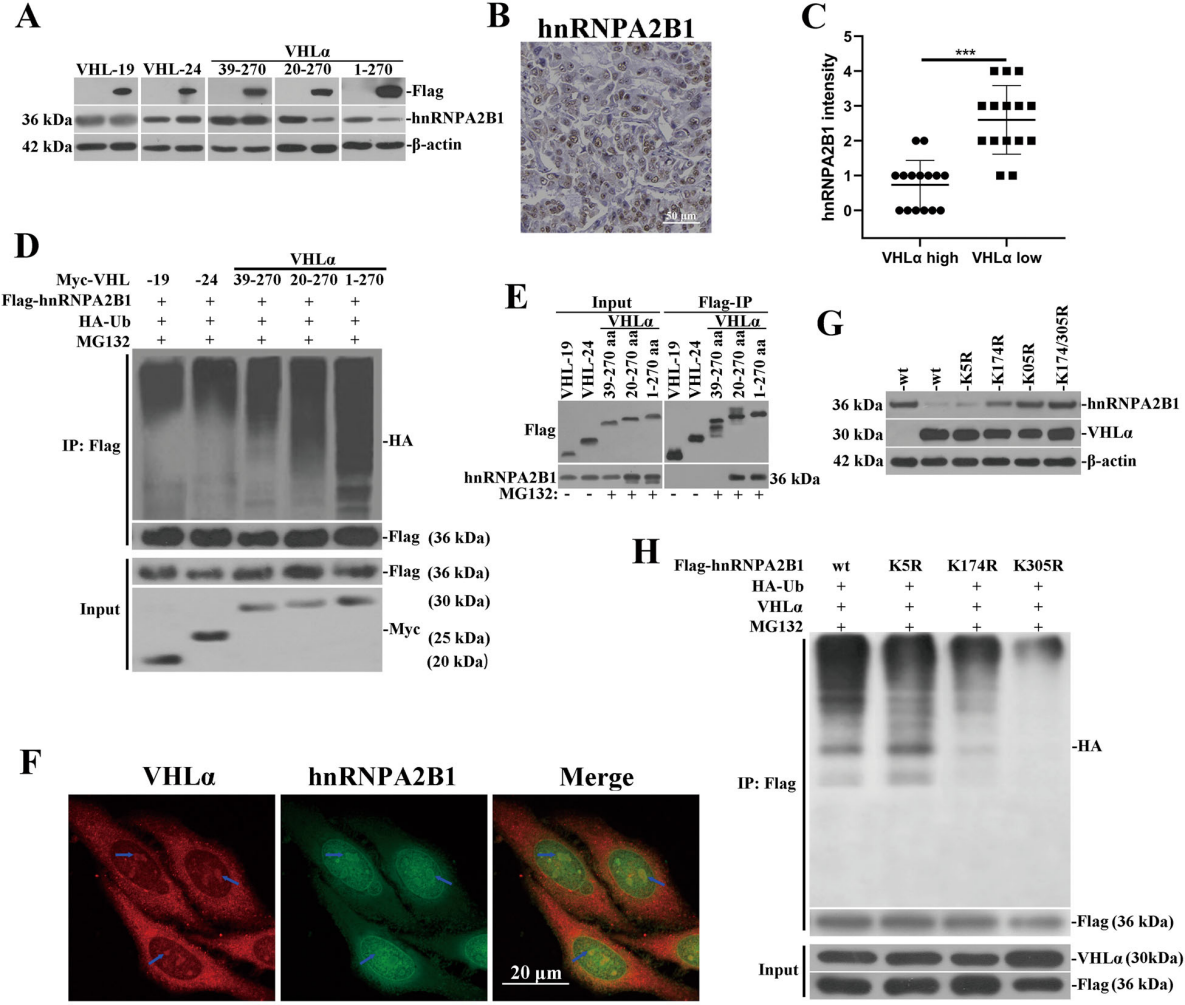
VHLα associates with and mediates ubiquitination/degradation of hnRNPA2B1. **a** Western blots analysis of endogenous hnRNPA2B1 protein in 293T cells with overexpression of indicated VHL transcripts. **b** Representative IHC images of renal tumors with positive staining signals of hnRNPA2B1. **c** IHC scoring correlation analysis of VHLα and hnRNPA2B1 in panel of renal tumors (*n* = 15 for each group). **d** Western blots with anti-HA antibody examining ubiquitination degree of hnRNPA2B1 in response to ectopic expression of indicated VHL fragments in presence of MG132. **e** Immunoprecipitation performed with anti-flag magnetic beads against cell lysates with overexpression of indicated VHL fragments and presence of hnRNPA2B1 was determined by western blots. MG132 was employed while transfected with VHLα. **f** Subcellular colocalization of VHLα and hnRNPA2B1 was investigated with confocal fluorescence microscope. **g** The potential impacts of indicated lysine-to-arginine mutations on hnRNPA2B1 degradation were evaluated by western blots in the context of intact VHLα transfection. **h** The potential impacts of indicated lysine-to-arginine mutations on hnRNPA2B1 ubiquitination were evaluated with anti-HA antibody. n.s., no significance; *P < 0.05; **P < 0.01; ***P < 0.001; ****P < 0.0001.

Our previous study demonstrated VHLα other than both VHL-19 and VHL-24 was capable of degrading hnRNPA2B1 in renal cancer cells. The serial truncate constructs manifested that 1–38 amino acid of VHLα was critical for degradation of hnRNPA2B1, as endogenous hnRNPA2B1 was significantly decreased by ectopic introduction of 20–270aa or 1–270aa VHLα but maintained unchanged in response to fragment 39–270 (Fig. 3a). Here we further showed the downregulation of hnRNPA2B1 in VHLα high-expressed renal tumor tissues, with representative IHC staining results of hnRNPA2B1 along with statistical results were provided in Fig. 3b, c. The immunoprecipitated hnRNPA2B1 was ubiquitinated to greater extent in the presence of MG132 while 293T cells were co-transfected with either full-length of VHLα or 20–270aa fragment in comparison with 39–270 fragment, VHL-19 or VHL-24, respectively (Fig. 3d). Consistently, we detected hnRNPA2B1 in immunoprecipitation complex against either full-length VHLα or 20–270 fragment (Fig. 3e). Our data suggested that the amino acid 1–38 of VHLα was critically for association with and ubiquitination of hnRNPA2B1. The direct interaction between hnRNPA2B1 and VHLα protein was further consolidated by the observation of partial subcellular co-localization in cell nuclear, which was highlighted by blue arrows in confocal images (Fig. 3f).

Next, we attempted to identify the ubiquitination site of hnRNPA2B1 by VHLα. Sequence analysis performed with UbPred algorithm (http://www.ubpred.org/) predicted that lysine residue-5, residue-174, and residue-305 as potential ubiquitination sites. We then employed mutagenesis PCR method to generate site-directed mutants of hnRNPA2B1 plasmids. Co-transfection with VHLα led to tremendous decrease of hnRNPA2B1 (wild-type and K5R), which was greatly canceled by K174R and K305R mutation individually or in combination (Fig. 3g). Consistently, the mutations at K174 and K305 other than K5 compromised the ubiquitination level of hnRNPA2B1 (Fig. 3h). Therefore, our data identified lysine residue 174 and 305 as ubiquitination sites by VHLα in renal cancer cells.

### c-myc positively regulated hnRNPA2B1 in renal cancer

**Fig. 4:**
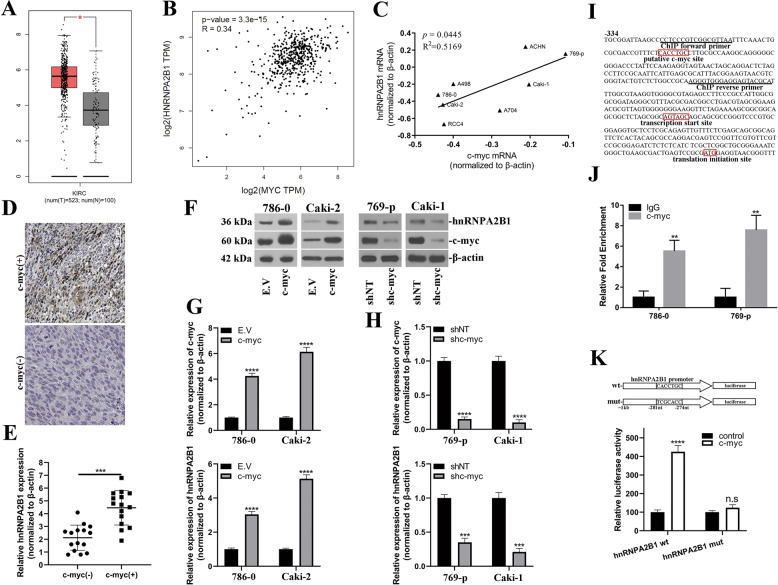
c-myc positively regulated hnRNPA2B1 in renal cancer. **a** Relative upregulation of c-myc in Kidney renal clear cell carcinoma (KIRC from TCGA) was analyzed with Gepia (http://gepia.cancer-pku.cn/). Tumors (n = 523) were compared to both matched TCGA normal samples and GTEX data (*n* = 100). **b** Correlation between c-myc and hnRNPA2B1 in KIRC was analyze with Gepia (*n* = 523, *R* = 0.34, *p* = 3.3e−15). **c** Relative expression of both c-myc and hnRNPA2B1 in our renal cancer cell line panel (*n* = 8) was determined by real-time PCR and correlation was analyzed (*R*^2^ = 0.5169, *p* = 0.0445). **d** Representative IHC images of c-myc(−) and c-myc(+) renal tumors. **e** The hnRNPA2B1 transcript abundance in renal tumors was analyzed by real-time PCR regarding c-myc intensity (*n* = 15 for each group). **f** Western blots analysis of hnRNPA2B1 in response to both c-myc overexpression (upper) and knockdown (lower). **g** Quantitative PCR determination of relative expression of both c-myc and hnRNPA2B1 in c-myc-proficient cells. **h** Quantitative PCR determination of relative expression of both c-myc and hnRNPA2B1 in c-myc-deficient cells. **i** Illustration of hnRNPA2B1 promoter region with putative c-myc recognizing site in red box. The ChIP primer sequences were underlined as well. **j** The direct binding of c-myc to hnRNPA2B1 promoter was investigated by ChIP assay and relative enrichment of corresponding fragment was measured by real-time PCR. **k** Luciferase reporter assay performed to interrogate the regulatory effects of c-myc on hnRNPA2B1. Either wild-type or putative recognizing site-mutated hnRNPA2B1 promoter-driven luciferase was co-transfected with control or c-myc-expressing plasmid for 24 h. n.s., no significance; *P < 0.05; **P < 0.01; ***P < 0.001; ****P < 0.0001.

Our previous data uncovered the self-limiting regulation of hnRNPA2B1 via feedback loop with VHLα in renal cancer cell. Here we sought to identify upstream regulatory mechanism at transcriptional level. Via datamining TCGA, we found significantly higher expression of c-myc in renal tumors and positive correlation with hnRNPA2B1 (Fig. 4a, b). This stringent correlation was validated by real-time PCR in our renal cancer cell line panel including 786–0, 769-p, Caki-1, Caki-2, ACHN, A498, A704, and RCC4 (Fig. 4c, *R*^2^ = 0.5169, *p* = 0.0445). We further analyzed relative abundance of hnRNPA2B1 in renal tumor tissues regarding c-myc status, and tremendous upregulation of hnRNPA2B1 transcripts were noticed in c-myc(+) tissues in comparison with c-myc(−) group (Fig. 4d, e). These data implicated a potentially regulatory effect of c-myc on hnRNPA2B1 transcription. Ectopic overexpression of c-myc greatly induced upregulation of hnRNPA2B1 at both protein and transcript levels, while shRNA-mediated knockdown of c-myc downregulated hnRNPA2B1 (Fig. 4f–h). With aid of bioinformatics tool (promo_v3), we predicted the putative c-myc site in promoter region of hnRNPA2B1 which was illustrated in Fig. 4i along with primer sequences used in ChIP assay. The significant enrichments of hnRNPA2B1 transcripts in immunoprecipitation complex with anti-c-myc antibody were observed in both 786–0 and 769-p cell (Fig. 4j). The regulatory effect of c-myc on hnRNPA2B1 transcription was further interrogated by luciferase reporter assay, wherein co-expression of c-myc greatly stimulated hnRNPA2B1 promoter-driven luciferase activities, which was readily abolished by scrambled mutation introduced into the putative c-myc-recognizing site (Fig. 4k). Therefore, our results clearly demonstrated hnRNPA2B1 transcription was subjected to c-myc in renal cancer cells.

### VHLα exerted anti-tumoral roles through negative control of oncogenic hnRNPA2B1 proteins in renal cancer

**Fig. 5:**
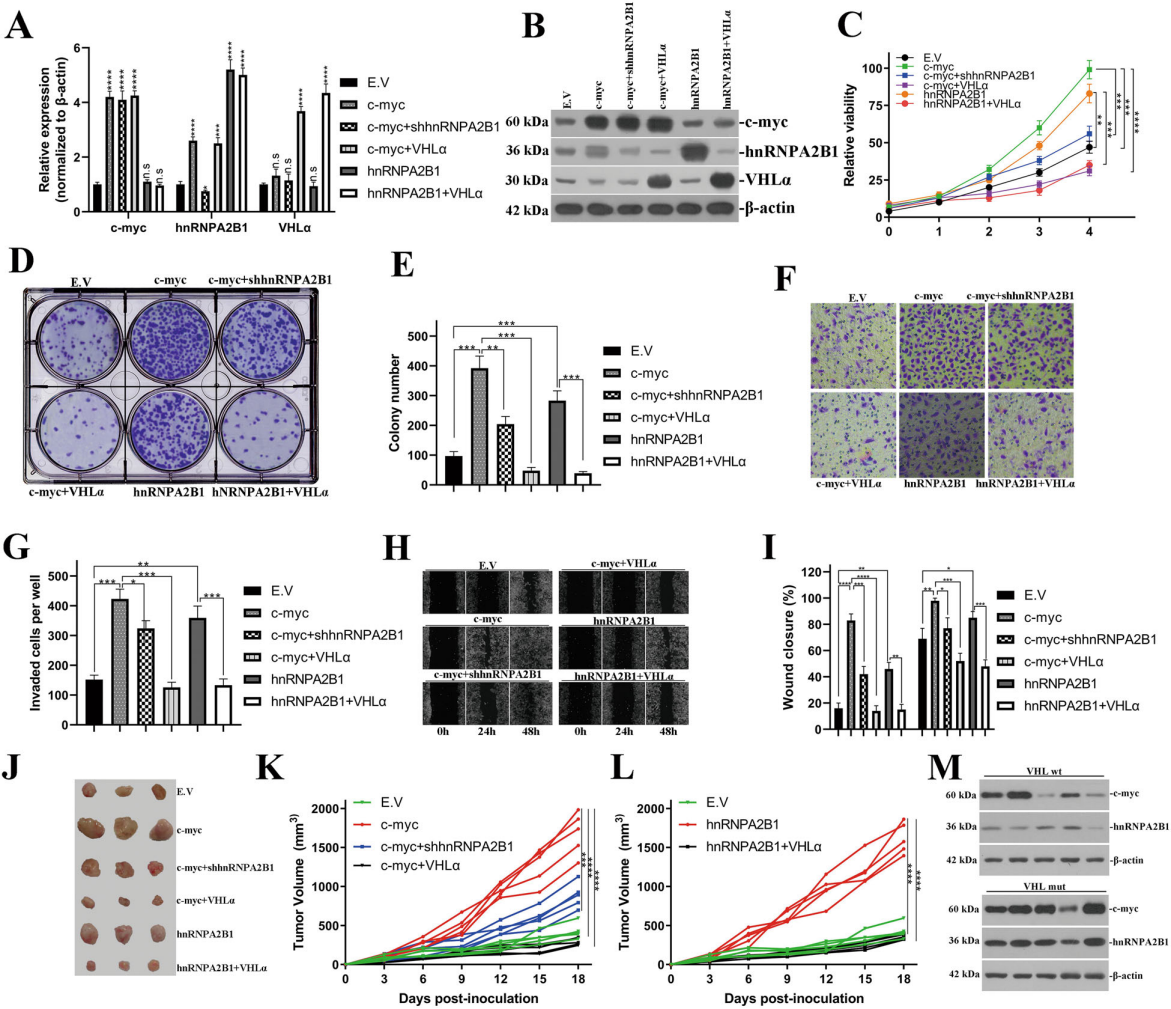
VHLα exerted anti-tumoral roles through negative control of oncogenic hnRNPA2B1 proteins in renal cancer cell. **a** Transcript analysis of c-myc, hnRNPA2B1 and VHLα in the indicated cells (empty vector, c-myc-overexpression alone or in combination with hnRNPA2B1 knockdown and VHLα-overexpression, hnRNPA2B1-overexpression alone or in combination with VHLα-overexpression) by real-time PCR. **b** Western analysis of c-myc, hnRNPA2B1 and VHLα proteins in cells as described in **a**. **c** Cell viability was determined with CCK-8 assay. **d** Representative images acquired from colony formation assay, and statistical results were provided in **e**. **f** Representative images of transwell assay employed to evaluate cell invasive capacities, and statistical results were provided in **g**. **h** Representative images of wound healing assay, and statistical results were provided in **i**. **j** Macroscopic images of xenograft tumors, and the corresponding xenograft tumor progression curve were plotted in panel **k** (empty vector, c-myc alone and in combination with either shhnRNPA2B1 or VHLα, *n* = 5 for each group) and **l** (empty vector, hnRNPA2B1 alone and in combination with VHLα, *n* = 5 for each group). **m** Western blots analysis of c-myc and hnRNPA2B1 in indicated ccRCC samples (wild-type and mutant VHL). n.s, no significance; *P < 0.05; **P < 0.01; ***P < 0.001; ****P < 0.0001.

Our previous results suggested that endogenous hnRNPA2B1 expression was modulated both by c-myc at transcriptional level and by VHLα through feedback at post-translational level. Here we sought to address the oncogenic properties of hnRNPA2B1 in the context of c-myc and VHLα. 786–0 cells were chosen for the following investigation in view of its intrinsic mutation of VHL and loss-of-function as E3 ligase to exclude potential interference of hnRNPA2B1-VHLα feedback loop. We first established stable cell with ectopic c-myc alone or in combination with either shhnRNPA2B1 or VHLα, and ectopic hnRNPA2B1 alone or in combination with VHLα. The success in establishments of stable cells were validated at both transcript level by quantitative PCR (Fig. 5a) and protein level by western blots (Fig. 5b). We noticed despite of high level of hnRNPA2B1 mRNA in c-myc and VHLα double-proficient cells, the protein level of hnRNPA2B1 was relatively low which was consistent with VHLα-mediated degradation mechanism. Ectopic introduction of c-myc significantly stimulated cell viability in comparison with empty control, which was greatly compromised by shRNA-mediated knockdown of hnRNPA2B1 (Fig. 5c) and further suppressed by co-transfection with VHLα. Likewise, introduction of hnRNPA2B1 alone was sufficient to upregulate cell viability, which was completely blockaded by VHLα. These results indicated the important roles of hnRNPA2B1 in mediating the pro-tumoral activities of c-myc, which was under stringent control by VHLα feedback loop. Notably, the greater inhibitory effects of VHLα than hnRNPA2B1-specific shRNA suggested the other anti-tumor mechanisms of VHLα such as inhibition of hypoxia induced factor as previously described^18^. The pro-proliferative activities of hnRNPA2B1 was also uncovered by colony formation assay, wherein c-myc overexpression dramatically increased the colony number which was greatly reduced by either hnRNPA2B1 depletion or VHLα-proficiency (representative images and statistical data were provided in Fig. 5d, e, respectively). Complementation with VHLα also completely inhibited colony formation stimulated by hnRNPA2B1 overexpression. The metastatic capacities were measured as well with both transwell assay and wound healing assay. As shown in Fig. 5f, g, c-myc significantly induced transwell capacities in comparison with empty vector control, which was partially inhibited by hnRNPA2B1 silencing while completely abolished by co-transfection with VHLα. Introduction of hnRNPA2B1 alone also promoted invasive behaviors while blockaded by VHLα. Consistently, the migrative measurement with wound healing demonstrated that ectopic c-myc significantly accelerated gap closure, which was slowed by hnRNPA2B1 silencing and further decelerated by VHLα. Transfection with hnRNPA2B1 alone imposed stimulatory effects on wound healing, and completely blockaded by ectopic VHLα (Fig. 5h, i). Most importantly, the oncogenic properties of hnRNPA2B1 downstream c-myc signaling and anti-tumoral feedback loop between hnRNPA2B1 and VHLα were consolidated with xenograft tumor in vivo. Forced overexpression of c-myc greatly promoted tumor progression, which was partially compromised by hnRNPA2B1 deficiency and completely abrogated by VHLα. HnRNPA2B1 proficiency alone also significantly contributed to xenograft tumor growth, and similarly inhibited by complementation of VHLα (macroscopic images of xenograft tumors were shown in Fig. 5j, and growth curve of xenograft tumor in the context of c-myc overexpression in combination with hnRNPA2B1 knockdown and ectopic VHLα was provided in Fig. 5k, growth curve of xenograft tumor in the context of hnRNPA2B1 overexpression in combination with VHLα overexpression was shown in Fig. 5l). We further analyzed functional relevance of VHLα, hnRNPA2B1 and c-myc in ccRCC tissue samples, and noticed relatively high abundance of hnRNPA2B1 in Myc+/VHLmt patients in comparison with Myc+/VHLwt ones (Fig. 5m). Our data demonstrated the oncogenic properties of hnRNPA2B1 especially in the context of c-myc signaling, and antagonized by its regulated VHLα.

## Discussion

Our previous study identified a novel alternative translation isoform of VHLα and preliminarily characterized its anti-tumor properties in renal cancer. Here we further unraveled its CUG-mediated initiation and subjection to both eukaryotic initiator factor eIF2A and RNA helicase eIF4A regulations. As expected, employments of ATG-initiating inhibitor ATA significantly induced VHLα translation, while CUT-initiating inhibitor acriflavine suppressed VHLα ratio at protein level. Via systematic knockdown method, we uncovered that eIF4A as the main RNA helicase involving in VHLα translation. Notably, we examined available eIF4A inhibitors including pateamine A, hippuristanol and silvestrol, all of which manifested significant inductions on VHLα translation and worthied further investigation into the potential anti-tumoral activities in this regard.

Our previous study also uncovered that VHLα negatively regulated hnRNPA2B1 and eventually modulated pyruvate kinase transcript splicing and reprogramed cellular glucose metabolism in renal cancer cells. Here we further identified the ubiquitination of hnRNPA2B1 at lysine residue 174 and 305 by VHLα, which critically depended on the amino acid 1–38 of VHLα. We speculated that this fragment which was unique to VHLα was responsible for both specificity and affinity to hnRNPA2B1, and therefore endowed VHLα other than VHL-24 and VHL-19 to mediate hnRNPA2B1 ubiquitination and degradation through proteasome pathway. Via retrieval of the Catalogue of Somatic Mutations in Cancer database (COSMIC, cancer.sanger.ac.uk/cosmic), we noticed that −134G > C point mutation locating in the N terminal of VHLα protein caused arginine to proline transition and therefore might disrupt interaction between VHLα and hnRNPA2B1 and VHLα-mediated hnRNPA2B1 degradation through ubiquitin-proteasome pathway. According to the subcellular localization as indicated by confocal images, VHLα-mediated ubiquitination of hnRNPA2B1 predominantly occurred in cell nucleus where hnRNPA2B1 exerted its primary biological function as heterogenous nuclear ribonucleoprotein.

Notably, in this study we unexpectedly found that VHLα other than VHL-19 and VHL-24 was transiently induced by ectopic hnRNPA2B1, which implicated the involvement of post-transcriptional mechanism in this scenario. In view of its intrinsic properties as RNA-binding protein, here we provided experimental evidence in support of the direct interaction between hnRNPA2B1 protein with VHL transcripts. We noticed both VHL-24 and VHLα were capable of complexing with hnRNPA2B1, which was absent in the VHL-19-pulldown protein species. The unusual octadic pentamer sequence predominantly mediated this interaction, and our results demonstrated that VHLα translation enhanced by ectopic hnRNPA2B1 was partially blockaded by mutation introduced into pentamer-5 and pentamer-8 both individually or in combination, and further completely abrogated by scramble mutation of whole octadic pentamer sequences. In this regard, we speculated that mutations curated in COSMIC database which located in pentamer region might manifest similar phenotype and abrogate the inducing effect of hnRNPA2B1 on VHL alternative translation. The delicate regulation and potential involvement of eIF4A in this scenario was still to be experimentally clarified.

The negative regulation of VHLα on hnRNPA2B1 via ubiquitination-proteasome degradation and positive regulation of hnRNPA2B1 on VHLα alternative translation herein constituted a feedback loop, which appeared to consequently contribute to self-limiting of hnRNPA2B1 in the context of upstream oncogenic signaling such as c-myc. In agreement with previous report, here we described c-myc-mediated regulation of hnRNPA2B1 transcription in renal cancer cells^26^. We would like to emphasize the distinct finding of our study in respect of c-myc-recognizing site in hnRNPA2B1 promoter in renal cancer. Via bioinformatic prediction and experimental validation, we identified −281~275 nt fragment as cis-element of c-myc and mutation of which greatly abolished the luciferase reporter activity of hnRNPA2B1. This regulation was further consolidated by the observation of significant positive correlation between c-myc and hnRNPA2B1 in kidney renal clear cell carcinomas curated in the TCGA database and in our renal tumor collection. Our data suggested upregulated hnRNPA2B1 as immediate events downstream aberrant c-myc signaling, which was subsequently subjected to the control of VHLα. Therefore, hnRNPA2B1 highly likely exert oncogenic functions in the context of c-myc activation and VHLα disruption in renal cancer. The current results suggested the specificity of VHLα exerting anti-tumoral function against activated hnRNPA2B1 in renal tumors, and therefore somatic mutations in VHLα which interrupted the feedback loop might compromise its tumor suppressor activity, which worthied further investigations to fully understand the novel isoform of VHL.

Our study also uncovered the pro-tumoral activities of hnRNPA2B1 in renal cancer cells. We found that introduction of c-myc tremendously stimulated cell proliferation, migration, invasion and xenograft tumor progression. This was in agreement with some previous reports. Tang et al. disclosed that myc signaling network was significantly activated and fundamental to the cell proliferation of clear cell renal cell carcinoma^27^. A conditional transgenic mouse model established by Shroff et al. demonstrated that myc oncogene overexpression was sufficient to initiate and maintain renal cell carcinoma, which critically upregulated glutaminolytic pathway instead of glycolytic pathway^28^. Our data suggested the oncogenic roles of c-myc was partially mediated by hnRNPA2B1 and greatly antagonized by VHLα, which was consistent with previous study showing that myc activation cooperated with VHL and INK4A/ARF loss in induction of clear cell renal cell carcinoma^29^. The insufficiency of myc in tumorigenesis in kidney with wild-type VHL implicated in this study was consolidated by our observations both in vitro and in vivo. We proposed that myc activation accelerated accumulation of hnRNPA2B1 in the context of VHL loss, which convergently contributed to the tumorigenesis. The tumorigenic activities of hnRNPA2B1 in VHL-deficient cells were also disclosed with stimulatory effects on cell proliferation, migration, invasion and xenograft tumor growth in our results, which was previously proposed by several investigations. For instance, Dowling et al. showed the abnormal levels of hnRNPA2B1 in solid tumors and peripheral blood from diagnosed lung cancer patients^30^. Through interaction with and regulation of oncogenic KRAS, hnRNPA2B1 was suggested involving in tumorigenesis of pancreatic ductal adenocarcinoma^31^. Therefore, our results unraveled a feedback loop between hnRNPA2B1 and VHLα, which exerted tumor suppressor roles downstream aberrant c-myc signaling.

In summary, our results demonstrated critical involvements of eIF2A and eIF4A in VHLα alternative translation, which specifically target hnRNPA2B1 to constitute a feedback loop and imposed stringent control on hnRNPA2B1 protein even in the context of c-myc aberrantly activated. In addition, we proposed therapeutic potentials of eIF4A inhibitors against renal cancer with intact VHL gene, which definitely worthied further investigations.

## Data Availability

All data generated or analyzed during this study are included in this published article.
